# Exploiting multi-granularity visual features for retinal layer segmentation in human eyes

**DOI:** 10.3389/fbioe.2023.1191803

**Published:** 2023-06-01

**Authors:** Xiang He, Yiming Wang, Fabio Poiesi, Weiye Song, Quanqing Xu, Zixuan Feng, Yi Wan

**Affiliations:** ^1^ School of Mechanical Engineering, Shandong University, Jinan, China; ^2^ Joint SDU-NTU Centre for Artificial Intelligence Research (C-FAIR), Shandong University, Jinan, China; ^3^ Fondazione Bruno Kessler, Trento, Italy

**Keywords:** multi-scale layer segmentation, deep learning, optical coherence tomography, NR206, ConvNeXt

## Abstract

Accurate segmentation of retinal layer boundaries can facilitate the detection of patients with early ophthalmic disease. Typical segmentation algorithms operate at low resolutions without fully exploiting multi-granularity visual features. Moreover, several related studies do not release their datasets that are key for the research on deep learning-based solutions. We propose a novel end-to-end retinal layer segmentation network based on ConvNeXt, which can retain more feature map details by using a new depth-efficient attention module and multi-scale structures. In addition, we provide a semantic segmentation dataset containing 206 retinal images of healthy human eyes (named NR206 dataset), which is easy to use as it does not require any additional transcoding processing. We experimentally show that our segmentation approach outperforms state-of-the-art approaches on this new dataset, achieving, on average, a Dice score of 91.3% and mIoU of 84.4%. Moreover, our approach achieves state-of-the-art performance on a glaucoma dataset and a diabetic macular edema (DME) dataset, showing that our model is also suitable for other applications. We will make our source code and the NR206 dataset publicly available at (https://github.com/Medical-Image-Analysis/Retinal-layer-segmentation).

## 1 Introduction

**FIGURE 1:**
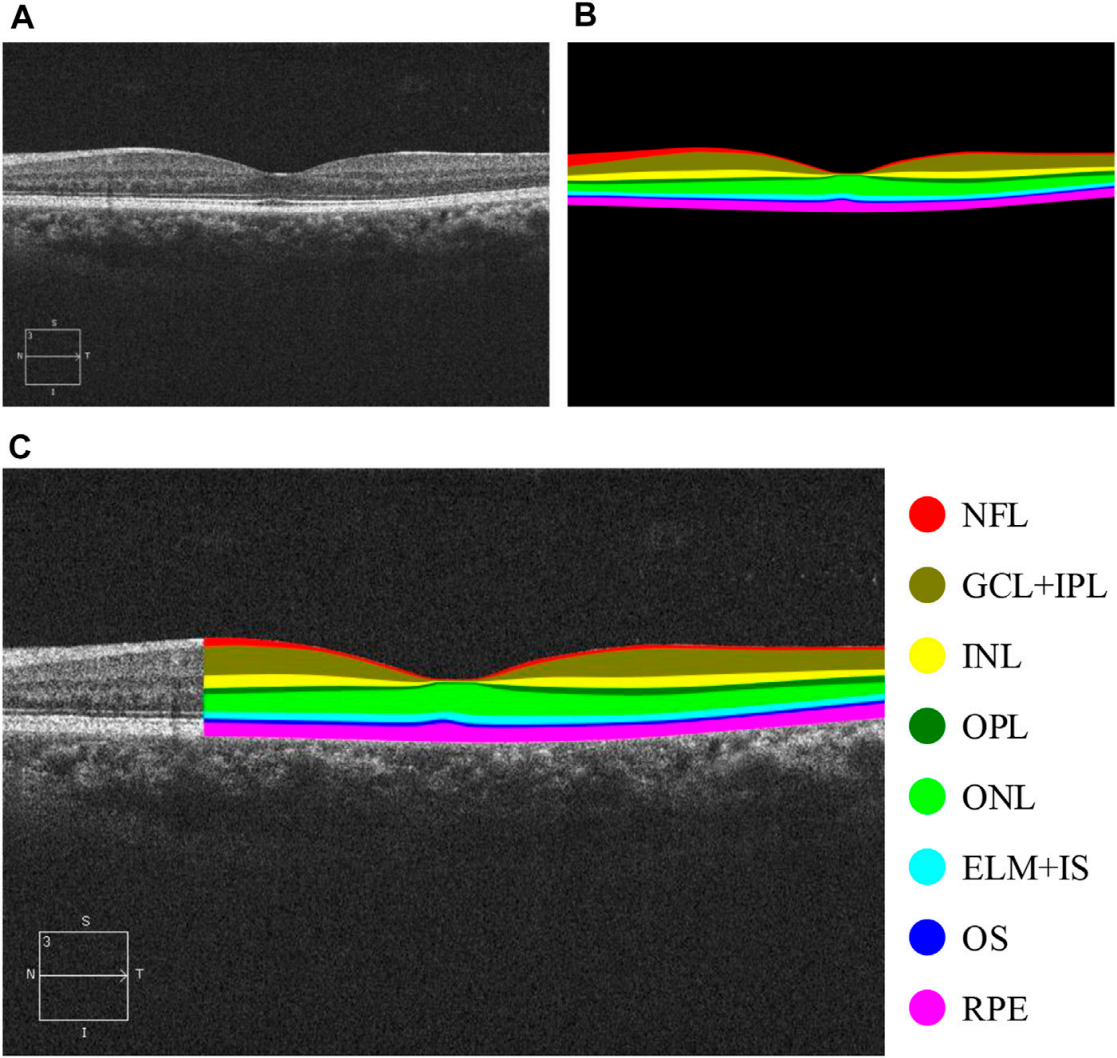
OCT B-scan images of retinal layers in healthy human eyes and annotations of each retinal tissue layer. **(A)** Original B-scan retinal layer image, **(B)** ground-truth, **(C)** eight classes of annotations for retinal layers, namely, NFL, GCL + IPL, INL, OPL, ONL, ELM + IS, OS, and RPE (Fernández et al., 2005), and other areas annotated as background class.

The retina is often referred to as the “window to the body,” as the early stages of many chronic diseases are associated with structural changes in the tissues of retinal layers (London et al., 2013; Pavkov et al., 2019). The precise quantification of changes in each retinal layer is a crucial step in monitoring disease progression (Ueda et al., 2022). Optical coherence tomography (OCT) is a non-invasive imaging technique that plays a pivotal role in clinical ophthalmology. It uses low-coherence interferometry to generate a two-dimensional image of internal tissue microstructures through optical scattering, akin to ultrasonic pulse-echo imaging (Huang et al., 1991). The imaging principle of OCT has evolved from time-domain OCT (TD-OCT) in 1991 (Huang et al., 1991) to spectral-domain OCT (SD-OCT) in 1995 (Fercher et al., 1995) and subsequently to swept-source OCT (SS-OCT) in 1997 (Chinn et al., 1997). OCT facilitates fundus retinal layer imaging (Huang et al., 1991), providing a clear representation of the structure of retinal layers and the thickness of each layer by leveraging differences in tissue structure and inter-layer distances (Figure 1). Since OCT images can directly and vividly display these characteristics, they are frequently employed as key indicators in the diagnosis of ophthalmic diseases (Ueda et al., 2022). Hence, by applying layer segmentation to OCT images of retinal layers, we can effectively monitor changes in the structure (thickness) of retinal layers.

Manual segmentation of retinal layers, traditionally an early method, has been labor-intensive, expensive, and challenging to standardize among different specialized ophthalmologists. Hence, automatic segmentation methods for retinal layers have become indispensable. Thanks to the rapid advancements in deep learning-based techniques, computational imaging approaches have become increasingly adept at automatically addressing this progression. These techniques rely on specialized datasets corresponding to the task to function reliably (Min et al., 2017). However, the retinal segmentation datasets employed in most studies are proprietary (Li et al., 2020). Only a few of the publicly available datasets can be used, and these require transcoding prior to application (Chiu et al., 2015; He et al., 2019). We contend that constructing a semantic segmentation dataset of the retinal layer of healthy human eyes is of significant importance.

Automatic retinal layer segmentation has significantly advanced in recent years. Several earlier methods utilized traditional computer vision techniques (Dufour et al., 2012; Tian et al., 2016; Karri et al., 2016), where layer segmentation was accomplished through multiple stages, including pre-processing tasks, such as denoising, and followed by post-optimization. However, these methods, due to their requirement for custom development for each unique problem, are being gradually supplanted by deep learning-based segmentation methods. Deep learning methods offer principal advantages for retinal layer segmentation tasks; they can autonomously extract image features and have the capability to generalize across diverse retinal images. ReLayNet (Roy et al., 2017) is a prevalent approach for retinal layer segmentation that operates on a U-Net framework (Ronneberger et al., 2015). However, the original U-Net framework has been superseded by attention-based frameworks that are capable of producing more informative and richer feature maps, thus resulting in improved segmentation outcomes (Chen et al., 2021). One major limitation of the U-Net framework is its lack of sufficient network depth. This deficiency results in the extraction of less informative feature maps, which consequently leads to diminished segmentation accuracy. Moreover, there exist deep learning-based methods that extract features at a smaller scale, which further limits the extent of informative features (Li et al., 2020).

In this paper, we introduce a novel, multi-scale, end-to-end deep learning model specifically designed for segmenting retinal layers in healthy human eyes using OCT B-scan images. Our solution addresses the limitations of current methods, which often fail to optimally preserve and utilize more detailed feature maps during segmentation. We achieve this by expanding the scale, reducing the upsampling multiple factors, and incorporating a depth-efficient attention module. Our strategy is built upon the latest ConvNeXt backbone. Additionally, we annotate and publicly release a dataset specifically for semantic segmentation of retinal layer OCT images in healthy human eyes. This dataset comprises 206 OCT B-scan images of healthy human eyes. Each image is categorized into nine classes, including eight retinal layers and the background, with an average of 14.82% of the pixels per image annotated as part of the retinal layers. We are confident that this newly created dataset will significantly contribute to the advancement of semantic segmentation methodologies in this field. Our semantic segmentation technique demonstrates superior performance on the NR206 dataset compared to existing methods, and we further corroborate its generalization capacity on two other publicly accessible datasets. The model and the dataset will be made available to the public upon the acceptance of this paper.

The organization of this paper is as follows: Section 2 presents the related work, covering both semantic segmentation methodologies and associated datasets. Section 3 outlines our proposed approach, detailing three main aspects: backbone networks, multi-scale feature encoding, and the depth-efficient attention module. Section 4 introduces our proposed NR206 dataset. Subsequently, in Section 5, we explain the experiments, interpret the model, and discuss the results. The final section concludes the paper and outlines future research directions.

## 2 Related work

### 2.1 Datasets

Several publicly accessible datasets of retinal layer optical coherence tomography (OCT) images currently exist online. Srinivasan et al. (2014) collected 45 sets of retinal images from 15 healthy subjects, 15 patients with dry age-related macular degeneration, and 15 patients with diabetic macular edema for disease classification. The study by Gholami et al. (2020) released an open-source database composed of four types of OCT images of ophthalmic diseases and one type of OCT images of healthy human eyes, encompassing more than 500 OCT B-scan images of human eyes. Chiu et al. (2015) extracted 110 B-scan images of the retinal layer from 10 patients with severe diabetic macular edema and manually segmented the boundaries of eight layers with the assistance of two ophthalmologists. Last, He et al. (2019) gathered OCT images from 14 healthy human eyes and 21 eyes of patients with multiple sclerosis, labeling images of the boundaries of nine layers. The majority of these publicly available datasets of retinal layer OCT images are primarily used for classification detection tasks and are not suitable for retinal layer segmentation. Moreover, some are compiled by MATLAB and need to be transcoded by professionals before use. Until 2017, retinal layer segmentation primarily used boundaries for layer segmentation. With the application of U-Net (Ronneberger et al., 2015) to medical segmentation tasks in 2015 and ReLayNet (Roy et al., 2017) to retinal layer segmentation in 2017, new annotation paradigms for layer segmentation of retinal layers have begun to emerge. Zhang et al. (2021) acquired a 3D scanned OCT image containing 273 eyes for 3D semantic segmentation of retinal layers. This pixel-level segmentation approach does not require consideration of the continuity of boundary lines, making it more universal compared to traditional threshold segmentation using boundary lines. However, most semantic segmentation datasets of retinal layers are not publicly available, which has hindered the advancement of the field.

### 2.2 Semantic segmentation methods

Since the introduction of the fully convolutional network (FCN) (Long et al., 2015), encoder–decoder architectures have been extensively used for a wide array of segmentation tasks. Notable networks, such as U-Net (Ronneberger et al., 2015), DeepLabV3+ (Chen et al., 2018), and UnetR (Hatamizadeh et al., 2022), all adopt this structure. Encoders generally leverage various backbone networks to extract highly semantic feature maps. With the swift advancement in multiple vision tasks, backbone networks have rapidly evolved in recent years. This includes CNN-based networks such as VGG (Simonyan and Zisserman, 2014), ResNet (He et al., 2016), Xception (Chollet, 2017), and ConvNeXt (Liu et al., 2022), as well as transformer-structured networks, such as ViT (Dosovitskiy et al., 2020) and Swin Transformer (Liu et al., 2021). The feature maps extracted by the backbone network are inputted into the decoder network, which contains an upsampling component, to restore the resolution of the image, thereby facilitating semantic segmentation.

Deep learning has made significant strides in the biomedical field (Le, 2021); (Le and Huynh, 2019). Since U-Net (Ronneberger et al., 2015) first applied semantic segmentation to medical applications, several dedicated semantic segmentation networks for the medical field have been proposed. These include Attention U-Net (Oktay et al., 2018), UnetR (Hatamizadeh et al., 2022), and FDK-Unet Wang et al. (2020). Furthermore, ever since ReLayNet (Roy et al., 2017) was first introduced for retinal layer segmentation, a surge in research in this task has been observed. For example, Li et al. (2021) proposed a two-stage network for segmenting the optic disc and retinal layers separately. The common goal of these works is to consistently optimize and enhance segmentation results, automate the segmentation of retinal layer OCT images, and advance the early diagnosis and treatment monitoring of retinal diseases.

DeepLabV3+ is a method developed by Chen et al. (2018), marking the final installment of the DeepLab series. This method utilizes the ASPP module to optimize the performance of dilated convolution. It adopts Xception as the backbone network (Chollet, 2017), combines encoder–decoder structures to design a new framework, and achieves state-of-the-art results on the PASCAL VOC 2012 and Cityscapes datasets (Chen et al., 2018). Despite its strengths, the model also has several limitations. First, the model only uses two scales of feature map information for upsampling restoration, namely, $F_{1}\in R^{C_{1}\times \frac{H}{4}\times \frac{W}{4}}$ and $F_{2}\in R^{C_{2}\times \frac{H}{16}\times \frac{W}{16}}$ . This not only involves fewer scales but also tends to lose detail with larger upsampling factors. Second, no weights are assigned to the feature map information at each scale, leading to inefficient use of the feature map information. Finally, the chosen backbone network has certain constraints, given that better-performing backbone networks have since been proposed.

## 3 Methods

### 3.1 Framework overview

**FIGURE 2:**
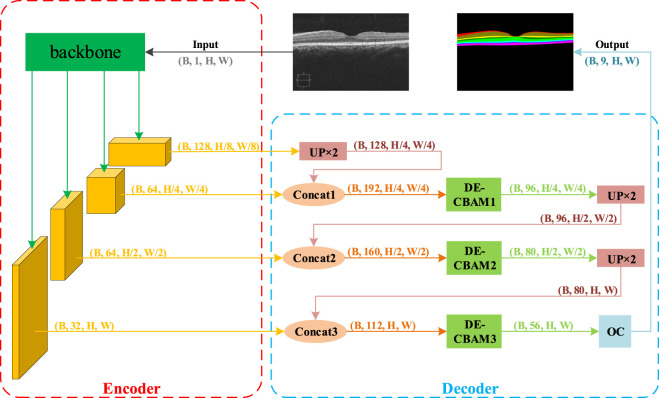
Overview of the proposed framework. This framework is composed of two primary components: the encoder and the decoder. During each training iteration, B retinal grayscale images are fed into the model. They first enter the backbone module of the encoder section for feature extraction, resulting in four feature maps. The feature map with the smallest size but the largest number of channels (B, 128, H/8, W/8) is then input into the first UP ×2 module for 2x upsampling restoration. The restored feature map’s size matches that of the adjacent output feature map from the backbone, allowing for channel concatenation. Following concatenation, the feature map is passed to the first DE-CBAM module for feature weighting. The output feature map, which has half the original channels but retains the same size, is then input into the second UP ×2 module. The subsequent steps mirror the previous ones. Finally, after exiting the third DE-CBAM block, the feature map is fed into the output convolution module, which adjusts the number of feature map channels to match the number of output target classes (nine classes in the NR206 dataset).

Our proposed methodology comprises an encoder module that extracts multi-scale features and a decoder module that amalgamates multi-scale information for upsampling reduction. Drawing inspiration from DeepLabV3+ (Chen et al., 2018), we utilize a modern backbone, ConvNeXt, to generate more informative visual embeddings. To address the problem of insufficient spatial details in the segmentation task, we introduce a more granular scaling of the feature map in the encoder section and corresponding upsampling processes in the decoder section. However, the incorporation of feature map resolutions at various granularities also compromises the computational complexity of the network. To address this, we propose a depth-efficient convolutional block attention module, abbreviated as DE-CBAM, within the decoder section to enhance the efficient utilization of feature information derived from the encoder. The structure of our proposed network is illustrated in Figure 2.

### 3.2 Backbone network

We employ the recently introduced convolutional network model, ConvNeXt (Liu et al., 2022), as our backbone for feature extraction. Floating-point operations per second (FLOPs) is a common metric used to quantify the computational cost. ConvNeXt addresses the FLOPs/accuracy trade-off using the concept of ResNeXt, with its central component being grouped convolution. This approach significantly reduces FLOPs, and the network width is expanded to compensate for the loss of capacity. To align with the non-local self-attention mechanism of the transformer, ConvNeXt employs a large kernel-sized convolution. This enhances the network’s performance while keeping the network’s FLOPs approximately constant. ConvNeXt has outperformed transformers in terms of accuracy, scalability, and robustness across most major benchmarks.

We carried out an extensive comparison among several backbone choices and empirically selected ConvNeXt for our feature extraction backbone. For more details, please refer to Section 5.4.

### 3.3 Multi-scale feature encoding

Multi-scale feature encoding involves extracting feature maps at various scales through the backbone, followed by fusion of the results via a series of concatenation or addition operations. Noting the limitation of the DeepLabV3+ model, which only utilizes feature map information at two scales for upsampling, we extend this to four scales. Theoretically, an increase in the number of scales enriches the information in the feature map. Additionally, the original DeepLabV3+ model uses a large upsampling factor, which is prone to detail loss. In addition, the upsampling reduction multiplier in the original DeepLabV3+ model is much larger and prone to loss of details, so we change the stem module of downsample_layers in ConvNeXt to change the downsampling from 4x to 1x, making the size of the output feature map change from $F_{1}\in R^{C_{1}\times \frac{H}{4}\times \frac{W}{4}}$ , $F_{2}\in R^{C_{2}\times \frac{H}{8}\times \frac{W}{8}}$ , $F_{3}\in R^{C_{3}\times \frac{H}{16}\times \frac{W}{16}}$ , and $F_{4}\in R^{C_{4}\times \frac{H}{32}\times \frac{W}{32}}$ to $F_{1}^{\prime }\in R^{C_{1}\times H\times W}$ , $F_{2}^{\prime }\in R^{C_{2}\times \frac{H}{2}\times \frac{W}{2}}$ , $F_{3}^{\prime }\in R^{C_{3}\times \frac{H}{4}\times \frac{W}{4}}$ , and $F_{4}^{\prime }\in R^{C_{4}\times \frac{H}{8}\times \frac{W}{8}}$ . The smaller the restoration multiplier is, the less the detailed information is lost in upsampling.

### 3.4 Depth-efficient convolutional block attention module

**FIGURE 3:**
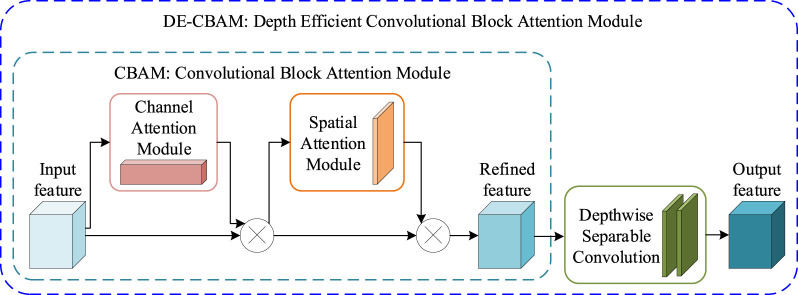
DE-CBAM module. The CBAM module is mainly composed of two sequential modules: CAM and SAM. The DE-CBAM module is plugged with two depth-separable convolutions into the output of the CBAM module.

The depth-efficient convolutional block attention module (DE-CBAM) employed in our proposed framework is based on an enhanced version of the convolutional block attention module (CBAM) (Woo et al., 2018). CBAM is an effective attention module for feedforward convolutional neural networks. Given an intermediate feature map, this module generates the attention map in sequence along two independent dimensions—channel and space—and then multiplies the attention map with the input feature map for adaptive feature refinement. Since CBAM is both lightweight and general-purpose, it can be seamlessly integrated into any CNN architecture with minimal overhead and can be trained end-to-end alongside the base CNN. Performance improvement across several classification and detection tasks when employing CBAM has been demonstrated (Woo et al., 2018). As illustrated in Figure 3, given an intermediate feature map $F\in R^{C\times H\times W}$ as input, CBAM successively infers a 1D channel attention map $M_{c}\in R^{C\times 1\times 1}$ and a 2D spatial attention map $M_{s}\in R^{1\times H\times W}$ . The overall attention process can be summarized as

$$F^{\prime }=M_{c}\left(F\right)⊗F,$$ (1)

$$F^{\prime \prime }=M_{s}\left(F^{\prime }\right)⊗F^{\prime },$$ (2)

where ⊗ denotes the element-wise multiplication and **F**″ is the final refined output. Channel attention first amalgamates the spatial information of a feature map using both average-pooling and max-pooling operations, generating two distinct spatial context descriptors: Favg^c^ and F max^c^. Both of these descriptors are then forwarded to a shared network to produce our channel attention map $F_{\max}^{\text{c}}$ , which is composed of a multilayer perceptron (MLP) with one hidden layer. After the shared network has been applied to each descriptor, the output feature vectors are finally merged using element-wise summation as

$$M_{c}\left(F\right)=\sigma \left(MLP\left(AvgPool\left(F\right)\right)+MLP\left(MaxPool\left(F\right)\right)\right).$$ (3)

The spatial attention module aggregates channel information of a feature map by using two pooling operations, generating two 2*D* maps: $F_{\mathrm{avg}}^{s}\in R^{1\times H\times W}$ and $F_{\max}^{s}\in R^{1\times H\times W}$ . Each denotes average-pooled features and max-pooled features across the channel. These are then concatenated and convolved by a standard convolution layer as

$$M_{s}\left(F\right)=\sigma \left(f^{7\times 7}\left(\left[AvgPool\left(F\right);MaxPool\left(F\right)\right]\right)\right),$$ (4)

where *σ* denotes the sigmoid function and *f* ^7 × 7^ represents a convolution operation with a filter size of 7 × 7.

We maintain the internal structure of the CBAM intact and, instead, introduce two consecutive depth-wise separable convolutions (Chollet, 2017) into its output section (Figure 3). Compared to conventional convolution, depth-wise separable convolution is able to increase network parameters with minimal impact, while ensuring that network depth is preserved. This modification not only increases the depth of the network but also enhances the utility of feature maps within the network, thereby improving overall network performance. Table 5 shows that our DE-CBAM module provides improvements over results obtained prior to the modification.

## 4 NR206 dataset

The NR206 dataset originates from 206 optical coherence tomography (OCT) retinal images of normal, healthy human eyes that are part of the OCTID (Gholami et al., 2020) database. Initially, these images were used for disease classification tasks along with five other retinal disease image sets. All the images are de-identified, maintaining the privacy of the patients involved. The images are taken using a raster scan protocol with a scan length of 2 mm. They were captured using a Cirrus HD-OCT machine at Sankara Nethralaya Eye Hospital in Chennai, India. An experienced clinical optometrist selected a fovea-centered image from each volumetric scan. The scans have an axial resolution of 5 *μ*m and a transverse resolution of 15 *μ*m (in tissue). The OCT machine uses a superluminescent diode with a wavelength of 840 nm as its optical source. The images are captured at a resolution of 500 × 750 pixels. We use this dataset for semantic segmentation, which allows us to study the variations in retinal layer thickness in healthy human eyes.

**FIGURE 4:**
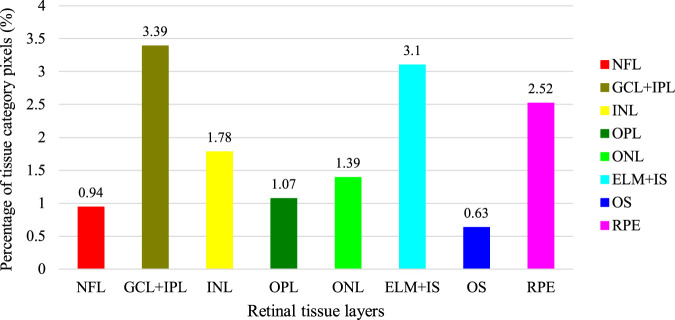
Average percentage of pixels on OCT images for each tissue layer in addition to the background (the percentage of the background is 85.18%).

Having obtained the required permissions from the original authors of the NR206 dataset, we embarked on a semantic segmentation annotation of these 206 retinal OCT images of healthy human eyes, guided by medical ophthalmology professionals. The process yielded a 10-class semantic segmentation dataset that includes a background class (refer to Figure 1). For annotation, we used a professional graphic software, Inkscape, and designated the eight retinal layers of NFL, GCL + IPL, INL, OPL, ONL, ELM + IS, OS, and RPE with different colors: red, brown, yellow, dark green, light green, light blue, dark blue, and pink, respectively. The remaining area was annotated as the background in black. Figure 4 presents the average pixel percentage across the OCT image for all classes excluding the background. It can be observed that the GCL + IPL and ELM + IS layers have larger pixel percentages, whereas the OS and NFL layers have smaller percentages. Following the annotation, each image was examined by a professional ophthalmologist and then exported as a 500 × 750 pixel PNG image. We divided the NR206 dataset into training, validation, and test sets comprising 126, 40, and 40 images, respectively.

## 5 Experiment

In order to evaluate our proposed approach, we tested it on our newly annotated NR206 dataset and two other public OCT datasets, in comparison with the recent state-of-the-art methods for semantic segmentation. An extensive ablation study is also presented to justify the design choices of our proposed method.

### 5.1 Experimental Setup

**Datasets.** The NR206 dataset, consisting of 206 OCT B-scan images of healthy human eyes, has been described previously. For each image, the size is 500 × 750. The dataset is partitioned into a training set of 126 images, a validation set of 40 images, and a test set of 40 images. Regarding the other two public datasets, 1) the glaucoma dataset (Li et al., 2021) contains images from 61 different subjects, with 12 radial OCT B-scans collected per subject at the Ophthalmology Department of Shanghai General Hospital using DRI OCT-1 Atlantis. Each image in this dataset has a size of 1,024 × 992. The dataset follows a training–validation–test split of 148, 48, and 48 images, respectively. (ii) The Duke SD-OCT dataset, which was collected by Chiu et al., comprises 110 OCT B-scans obtained from 10 patients with diabetic macular edema. Each image in this dataset has a size of 496 × 768 pixels. More details about this dataset can be found in the study by Chiu et al. (2015).

**Performance metric.** To account for class imbalance in our datasets, we use the Dice score, mIoU, Acc, and mPA for quantitative evaluation of segmentation performance. They are formulated as follows:

$$Dice=\frac{2TP}{2TP+FP+FN},$$ (5)

$$mIoU=\frac{1}{k+1}\overset{k}{\underset{i=0}{\sum }}\frac{TP}{FN+FP+TP},$$ (6)

$$Acc=\frac{TP+TN}{TP+TN+FP+FN},$$ (7)

and

$$mPA=\frac{1}{k+1}\overset{k}{\underset{i=0}{\sum }}\frac{TP}{TP+TN+FP+FN},$$ (8)

where *TP* represents true positives, where the predictions match the ground-truth labels, *TN* represents true negatives, where the predictions and ground-truth labels are both negative, *FP* represents false positives, where the predictions are positive but ground-truth labels are negative, *FN* represents false negatives, where the predictions are negative but ground-truth labels are positive, and *k* represents the number of categories.

**Implementation details.** Our method is implemented using PyTorch, and the model is trained on NVIDIA GeForce RTX 3090. We keep the training batch size the same for all the methods, determined by the capacity of the GPU, and utilize k-fold cross-validation on the training set; specifically, we employ 4-fold cross-validation. We adopt the Adam optimizer and use a StepLR scheduler for adjusting the learning rate during training. The network is trained using the cross-entropy loss function. In order to augment the dataset size, we apply data augmentation techniques, including horizontal flipping, random rotation, additive blur, and contrast adjustment. Training is set to stop automatically after 300 epochs, and the model weights that deliver the best performance on the validation set are chosen for testing. For the NR206 dataset, we set the learning rate to 0.002 with the image size cropped to (480,736) and conduct 4-fold cross-validation experiments on it. For the other two public datasets, we set the image size to (608,608) for the glaucoma dataset (Li et al., 2021) and (384,480) for the DME dataset (Chiu et al., 2015), respectively.

### 5.2 Comparison

We compare our proposed approach with several state-of-the-art methods for image segmentation tasks, including DeepLabV3+ (Chen et al., 2018), Attention U-Net (Oktay et al., 2018), ReLayNet (Roy et al., 2017), OS_MGU (Li et al., 2021), BiSeNet (Yu et al., 2018), and UnetR (Hatamizadeh et al., 2022).

**FIGURE 5:**
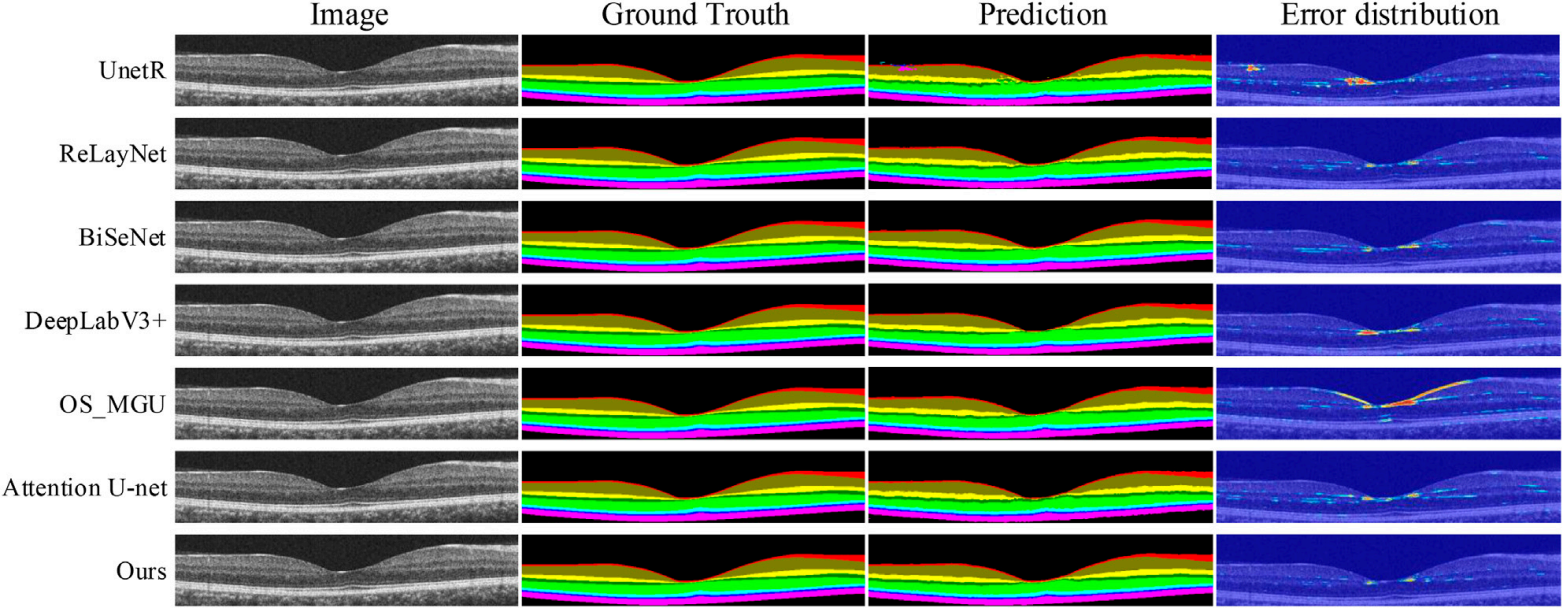
Retinal layer segmentation prediction graphs and error distribution maps on one random retinal test image in the NR206 dataset for each method.

Table 1 presents the performance comparison of our method with other state-of-the-art methods on the NR206 dataset. Overall, our approach outperforms the compared methods across most of the layers, resulting in the highest average Dice score, mIoU, Acc, and mPA, improving upon the second-best method by +0.8% in mIoU and +0.4% in Acc. Specifically, our method surpasses OS_MGU in terms of mIoU and Acc by +1.0% and +0.7%, respectively. We further perform a statistical significance test, using the Wilcoxon rank-sum test, to compare the Dice score performance of different methods on each layer (Lam and Longnecker, 1983). For instance, when comparing our method with RelayNet on the same domain, we observe a *p-*value of 0.017960 (p < 0.05), indicating a statistically significant difference. A similar statistically significant difference is observed with a *p-*value of 0.007812 (p < 0.05) when comparing our method with OS_MGU. Although DeepLabV3+ (Chen et al., 2018) is a widely recognized method for semantic segmentation, performing well in many scenarios, its segmentation accuracy on our dataset is found to be lower than our proposed method across all retinal tissue layers, with the mIoU metric being 1.3% lower. Our analysis suggests that the key reason for this performance disparity is that DeepLabV3+ only utilizes two scales for feature extraction, which could lead to the neglection of some detailed information during feature map extraction. Furthermore, the coarse feature maps generated by the 4-fold upsampling operation in DeepLabV3+ may result in accuracy loss. The prediction map of DeepLabV3+ in Figure 5 indicates discontinuities in several layers within the macular region. It is known that the retinal layer thickness is thinnest in the macular region of the retina (Chan et al.,2006); therefore, each tissue layer should be continuous and without disruptions. Moreover, the error distribution map of DeepLabV3+ demonstrates significant boundary inaccuracies occurring at the boundary between the OPL layer and the ONL layer outside the macular area.

In contrast, our method is capable of achieving more detailed and accurate segmentation of the macular retinal layers, while maintaining continuity of the tissue layers, due to the multi-scale feature extraction and reduced upsampling factors in our approach. ReLayNet (Roy et al., 2017) is an important work in retinal layer segmentation, using a multi-scale network architecture based on the U-Net (Ronneberger et al., 2015). However, as shown in Table 1, ReLayNet falls short in performance when compared to our method on all retinal layers. Figure 5 shows that ReLayNet generates less smooth boundaries than our method, with the boundary inaccuracies further highlighted by the error distribution maps. Our analysis suggests that the multi-scale network structure of ReLayNet does not fully leverage its multi-scale feature maps. Our method, on the other hand, applies the DE-CBAM structure, specifically designed for each level of the feature map, thereby improving the efficiency of feature utilization at every level. Furthermore, as shown in Figure 5, prediction maps of UnetR (Hatamizadeh et al., 2022) reveal significant segmentation errors in the GCL layer, as well as visible errors in the macular area. Similarly, BiSeNet (Yu et al., 2018) and Attention U-Net (Oktay et al., 2018) exhibit varying degrees of segmentation errors in the macular layer.

**TABLE 1:** Dice score (%), mIoU (%), Acc (%), and mPA (%) of the segmentation results on the NR206 dataset by different methods. The best result is highlighted in bold.

Method	Dice score									mIoU	Acc	mPA
	NFL	GCL + IPL	INL	OPL	ONL	ELM + IS	OS	RPE	Average			
UnetR	87.4 ± 0.7	94.2 ± 0.5	87.1 ± 0.7	77.5 ± 1.0	93.8 ± 0.4	91.3 ± 0.4	87.3 ± 0.4	95.3 ± 0.2	89.2 ± 0.4	81.0 ± 0.7	89.2 ± 0.7	98.4 ± 0.0
ReLayNet	88.7 ± 0.5	95.8 ± 0.1	90.1 ± 0.3	81.6 ± 0.2	95.2 ± 0.1	92.2 ± 0.4	87.5 ± 0.0	**96.1** ± 0.1	90.9 ± 0.1	83.6 ± 0.1	91.0 ± 0.2	98.7 ± 0.0
BiSeNet	88.7 ± 0.5	96.0 ± 0.1	**90.8** ± 0.1	81.8 ± 0.5	95.0 ± 0.1	91.5 ± 0.4	86.2 ± 0.7	96.0 ± 0.1	90.8 ± 0.2	83.4 ± 0.3	90.7 ± 0.5	98.7 ± 0.0
DeepLabV3+	88.2 ± 1.4	95.7 ± 0.2	90.0 ± 0.1	81.3 ± 0.8	95.1 ± 0.2	92.4 ± 0.2	86.3 ± 1.4	95.5 ± 0.4	90.6 ± 0.6	83.1 ± 0.7	90.5 ± 1.1	98.7 ± 0.0
OS_MGU	88.8 ± 0.7	95.8 ± 0.3	90.1 ± 0.5	80.9 ± 0.7	94.9 ± 0.2	92.2 ± 0.3	87.4 ± 0.1	96.0 ± 0.2	90.7 ± 0.3	83.4 ± 0.5	90.7 ± 0.4	98.7 ± 0.0
Attention U-Net	87.7 ± 0.7	95.3 ± 0.3	89.1 ± 0.3	80.4 ± 0.8	94.7 ± 0.3	92.0 ± 0.3	86.5 ± 1.7	95.8 ± 0.2	90.2 ± 0.3	82.5 ± 0.4	89.9 ± 0.6	98.6 ± 0.1
Ours	**89.7** ± 0.4	**96.1** ± 0.1	90.5 ± 0.2	**82.4** ± 0.2	**95.3** ± 0.1	**92.7** ± 0.1	**87.7** ± 0.3	**96.1** ± 0.1	**91.3** ± 0.1	**84.4** ± 0.2	**91.4** ± 0.3	**98.8** ± 0.0

**FIGURE 6:**
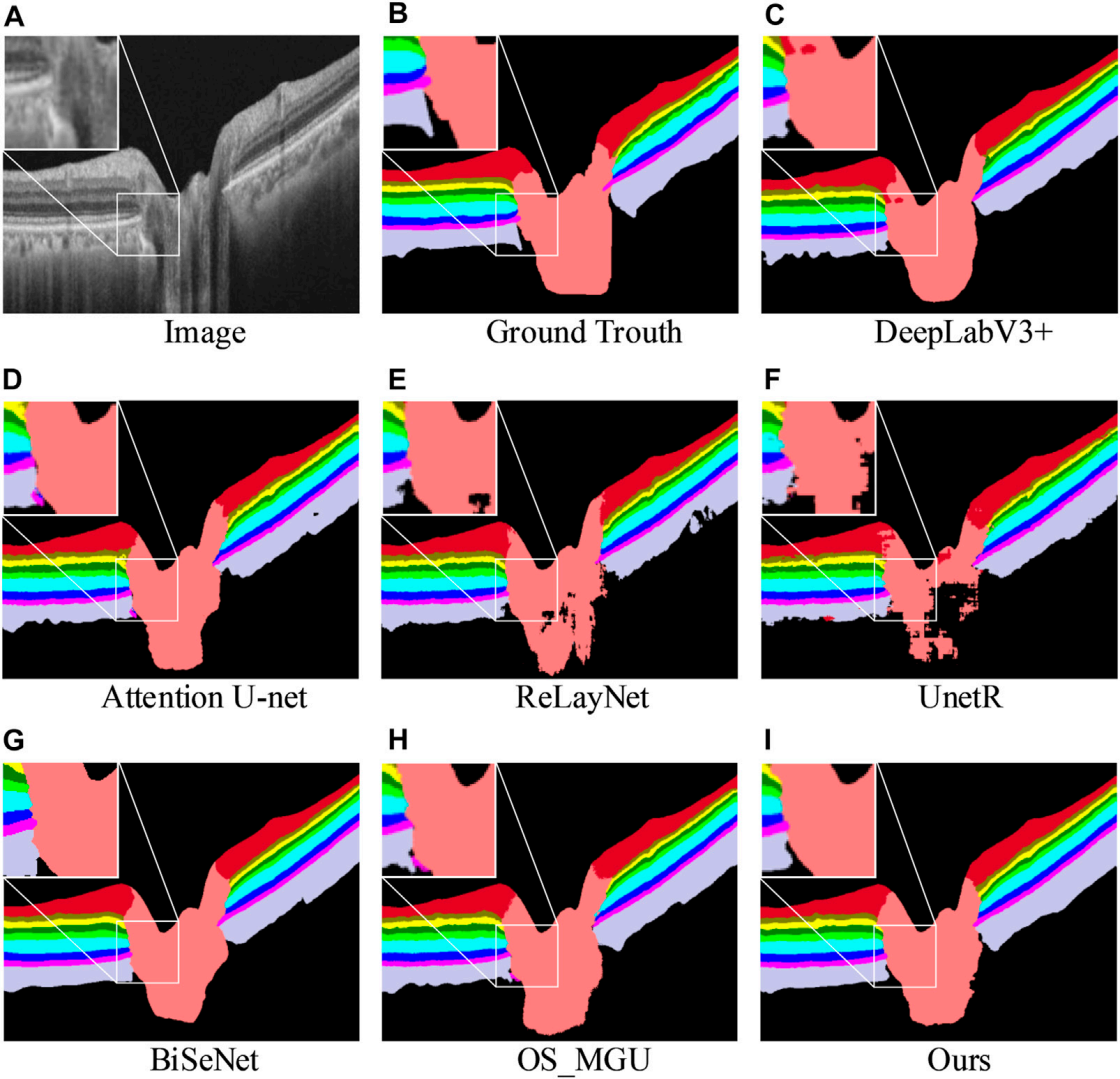
Retinal layer segmentation prediction graphs on one random retinal test image from the glaucoma dataset **(A)** for the original image, **(B)** for the ground-truth, **(C)** for the prediction of DeepLabV3+, **(D)** for the prediction of Attention U-Net, **(E)** for the prediction of ReLayNet, **(F)** for the prediction of UnetR, **(G)** for the prediction of BiSeNet, **(H)** for the prediction of OS_MGU, and **(I)** for the prediction of our method. An enlarged graph of the local details is also given in the prediction image for each method.

Table 2 shows the segmentation results of various methods on the dataset (Li et al., 2021), which includes the optic nerve head region. It is imperative to perform segmentation in this region as well, given that early stages of numerous diseases have been linked with optic nerve head atrophy and changes in the outer retina layer area (Bhartiya et al., 2010; Chrástek et al., 2005). Given the complex morphology of the retinal biological tissue in the optic nerve head area, performing layer segmentation in this region is also an essential step in the evaluation of retinal layer models. According to the results, our approach achieves the highest Dice score, mIoU, Acc, and mPA for most of the semantic classes, except for the OPL, ONL, IS + OS, and choroid classes. However, when considering the average Dice score, our approach achieves the highest segmentation accuracy, surpassing the second-best method by +0.9%. Furthermore, our method outperforms OS_MGU in terms of mIoU and Acc metrics by +1.3% and +1.9%, respectively. One additional advantage of our model is its smaller size in terms of parameters. Specifically, when compared with Attention U-Net and DeepLabV3+, our model only has 0.05 times and 0.03 times their respective parameter sizes, demonstrating the efficiency of our model. Figure 6 provides a comparison of the retinal layer segmentation performance of different methods in the optic disc (OD) area, illustrating the superior segmentation capability of our model. As depicted in the magnified view in Figure 6C, the DeepLabV3+ method introduces segmentation errors, incorrectly identifying the NFL layer as OD tissue. Similarly, as shown in the magnified view in Figure 6H, the OS_MGU method incorrectly classifies the background region as RPE layer tissue. Both ReLayNet and UnetR also display varying degrees of segmentation errors in the OD layer. These results collectively emphasize the robustness and precision of our proposed model in challenging retinal layer segmentation tasks.

**TABLE 2:** Dice score (%), mIoU (%), Acc(%), and mPA (%) of the segmentation results on the glaucoma dataset (Li et al., 2021) by different methods. The best performance is in bold.

Method	Dice score											mIoU	Acc	mPA	Params (M)
	RNFL	GCL	IPL	INL	OPL	ONL	IS + OS	RPE	Choroid	OD	Average				
UnetR	80.5	62.6	68.1	73.1	78.1	88.6	84.5	82.0	85.9	75.7	78.4	64.5	76.9	93.8	76.2
ReLayNet	79.3	65.5	70.9	76.9	**81.2**	**90.4**	**86.0**	81.7	87.8	79.3	80.3	67.1	79.4	94.3	0.7
BiSeNet	79.9	63.5	70.0	73.3	78.3	89.2	83.9	80.2	88.0	83.3	79.4	65.9	79.3	94.7	13.1
DeepLabV3+	79.9	63.9	68.5	75.1	78.7	88.8	83.3	79.5	87.9	82.7	79.3	65.7	78.6	94.6	54.6
OS_MGU	80.8	61.7	70.6	76.3	80.2	**90.4**	85.9	81.7	**88.7**	84.0	80.6	67.5	79.6	95.0	2.0
Attention U-Net	80.4	60.3	69.0	75.1	78.0	90.3	85.9	82.4	88.1	83.2	79.9	66.5	79.7	94.7	34.8
Ours	**80.9**	**67.1**	**73.8**	**77.4**	80.8	89.9	85.5	**82.8**	88.6	**84.4**	**81.5**	**68.8**	**81.5**	**95.1**	1.9

**FIGURE 7:**
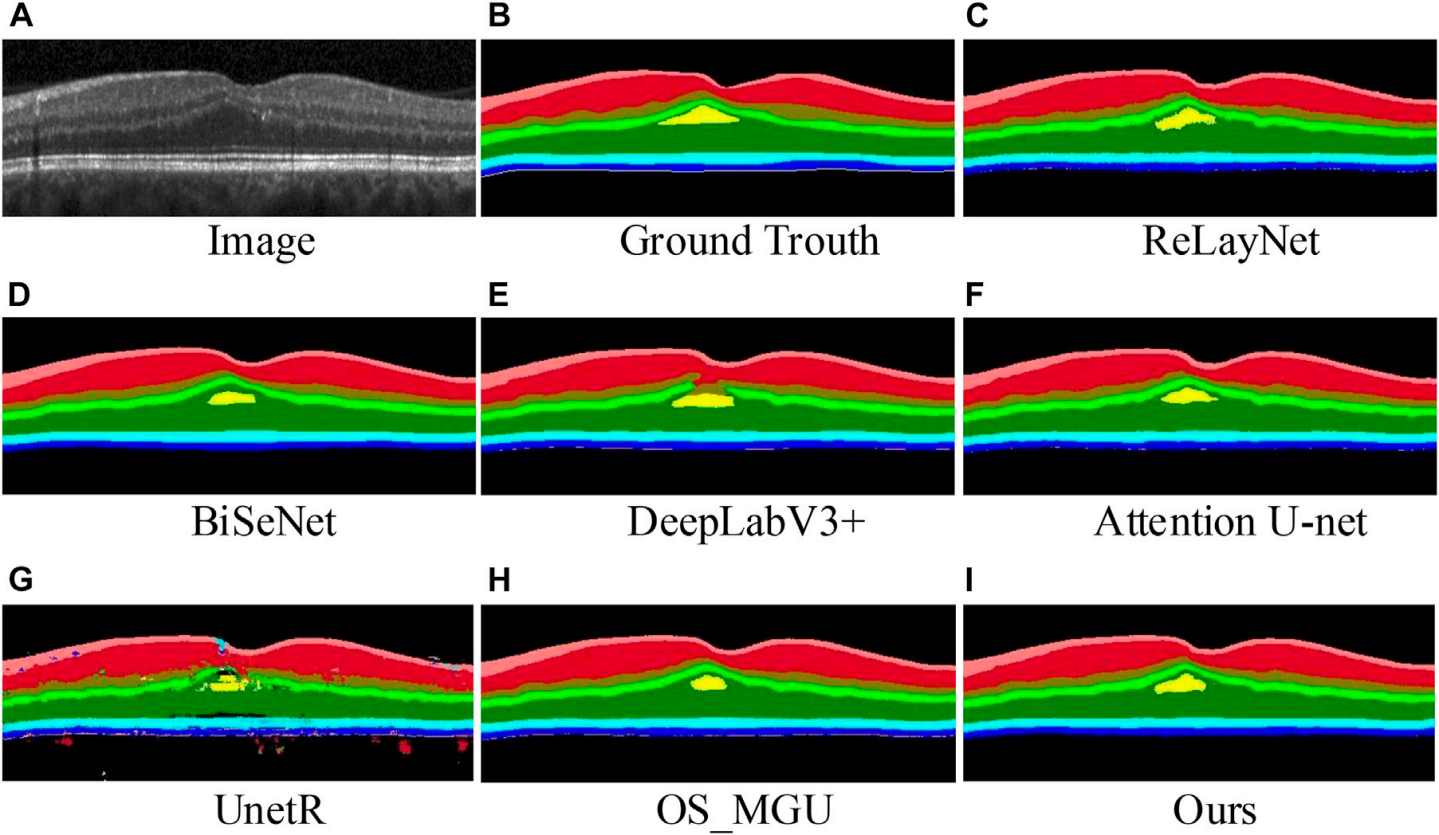
Retinal layer segmentation prediction graphs on one random retinal test image from the DME dataset **(A)** for the original image, **(B)** for the ground-truth, **(C)** for the prediction of ReLayNet, **(D)** for the prediction of BiSeNet, **(E)** for the prediction of DeepLabV3+, **(F)** for the prediction of Attention U-Net, **(G)** for the prediction of UnetR, **(H)** for the prediction of OS_MGU, and **(I)** for the prediction of our method.

The segmentation of retinal layers in retinal images without significant structural changes in the health or retinal layer is generally straightforward. However, many ophthalmic diseases, such as diabetic retinopathy (Stitt et al., 2016), can cause considerable alterations in the structure of the retinal layer. Therefore, segmenting retinal layers in images depicting such disease conditions is a more challenging task and provides a comprehensive test of the model’s capability. Table 3 compares our method with other methods on a dataset of patients suffering from DME (Chiu et al., 2015). The results show that our approach outperforms the other methods, surpassing the second-best method by +0.9% in terms of mIoU and +1.1% in terms of Acc. Our method shows superior segmentation capabilities in most layers, particularly in the lesion fluid layer, where it achieves the best results, leading the second-best method by +2.7% in terms of the Dice score. Figure 7 shows that images (e) and (g) contain evident segmentation errors, and the edge of the fluid layer in image (c) has visible serrations. However, our method not only achieves effective segmentation of the retinal layer but also outperforms the other methods in the segmentation of the lesion fluid layer. These results highlight the robustness and precision of our model in the challenging task of retinal layer segmentation under disease conditions.

**TABLE 3:** Dice score (%), mIoU (%), Acc(%), and mPA (%) of the segmentation results on the DME dataset (Chiu et al., 2015) by different methods. The best performance is in bold.

Method	Dice score									mIoU	Acc	mPA
	NFL	GCL	INL	OPL	ONL	ISE	OS	Fluid	Average			
UnetR	70.6	81.2	63.5	63.3	80.6	80.0	77.3	38.6	70.6	54.6	68.1	91.5
ReLayNet	81.1	93.1	78.5	76.9	87.1	**86.9**	86.2	59.0	81.8	69.3	80.1	95.3
BiSeNet	**81.7**	**93.2**	78.6	77.8	85.8	86.1	85.2	55.6	81.3	68.5	79.0	95.4
DeepLabV3+	80.3	92.2	76.7	75.8	85.9	86.8	86.5	55.4	80.8	67.8	79.5	95.3
OS_MGU	81.6	93.1	79.4	**79.4**	86.6	86.3	86.3	55.6	81.9	69.3	80.0	95.5
Attention U-Net	80.2	91.5	77.7	76.3	87.1	86.5	85.8	54.3	80.8	67.8	79.0	95.0
Ours	**81.7**	93.1	**80.2**	77.5	**87.2**	**86.9**	**86.6**	**61.7**	**82.5**	**70.2**	**81.2**	**95.7**

While our model achieves the best segmentation performance on all three datasets and maintains a relatively low model parameter count, it does entail a somewhat higher computational cost. In our experiments, the average inference time on a CPU was 2.04 s for the test images of the NR206 dataset and 1.00 s for those of the DME dataset. This higher computational cost is primarily due to the model’s intricate structure, which incorporates a multi-scale feature extraction structure aimed at harnessing multi-granularity visual features. This complexity leads to increased memory usage and a greater number of operations. In real-world clinical settings, most medical equipment may not meet the high hardware requirements, such as high-performance GPUs, that are available during the model development stage. Consequently, in addition to delivering high-accuracy segmentation results, segmentation models also need to focus on enhancing the efficiency of image inference. This would make such models more practically applicable, particularly in resource-constrained settings. Future work will thus aim to optimize the trade-off between model complexity and computational cost, ensuring the model remains accurate while also becoming more efficient.

### 5.3 Model interpretation

**FIGURE 8:**
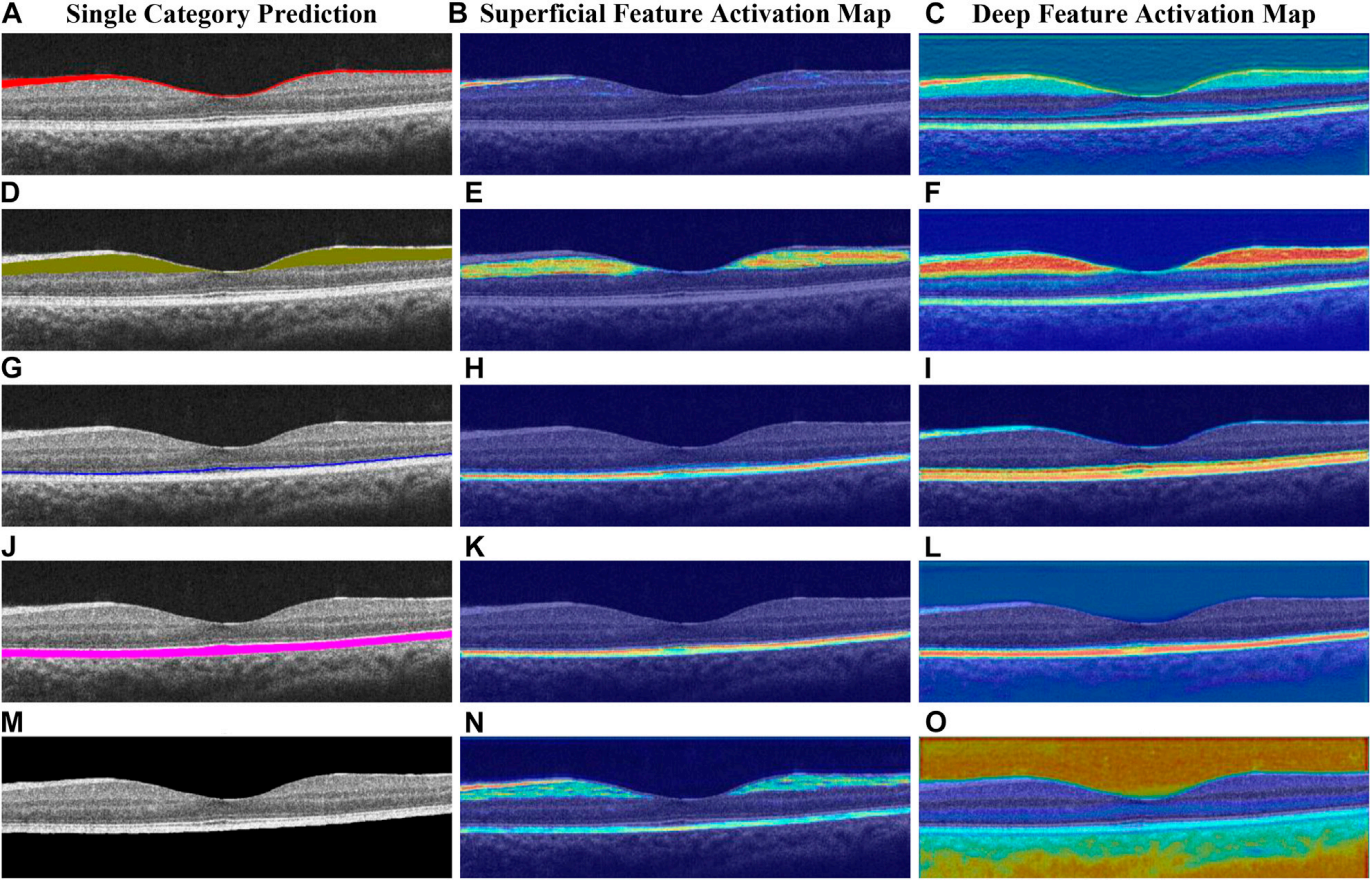
Heatmap of the deep network layer and superficial network layer obtained by different categories, respectively, **(A–C)** for the NFL, **(D–F)** for the GCL + IPL, **(G–I)** for the OS, **(J–L)** for the RPE, and **(M–O)** for the background.

To interpret the output of our model, we utilize a modified version of Grad-CAM (Vinogradova et al., 2020) to visualize feature activations at different layers of the network (see Figure 8). Specifically, we extract the feature activations from DE-CBAM1 (early layer) and the OC block (deep layer). In Figure 8, the heatmap generated by DE-CBAM1 exhibits edge-like structures. The activated region in the heatmap (Figure 8E) bears a resemblance to the outline of the prediction map for that specific layer shown in Figure 8D. This observation indicates that the superficial network primarily captures low-level image features. Furthermore, the activated region in the heatmap (Figure 8H) roughly corresponds to the upper and lower boundaries of the OS layer. This finding aligns with the notion that the deeper layers of the network capture higher-level features and contextual information. By visualizing the feature activations at different layers, we gain insights into the model’s decision-making process and understand which image regions contribute more strongly to the segmentation results.

Upon analyzing the deep feature activation map, we observe that not only are the regions corresponding to the prediction layer prominently highlighted but the feature weights of adjacent layers are also significantly amplified. This phenomenon suggests that the model leverages information from neighboring layers to enhance its predictions. For instance, in Figure 8F, the focus is primarily on the GCL layer, but the neighboring layers, such as NFL and INL, also exhibit varying degrees of activation. Similarly, in Figure 8O, the background area of the retinal layer is the main focus, while information from the NFL and RPE layers at the upper and lower boundaries of the retinal layer is also utilized. To understand this behavior, we conducted further analysis and found that the boundaries of retinal layers exhibit a high degree of correlation with adjacent layers. This observation suggests that the deeper layers of the model network extract image features that contain richer and more insightful semantic information. By incorporating information from neighboring layers, the model improves its ability to capture the fine details and contextual information necessary for accurate segmentation. Overall, the visualization of deep feature activations provides valuable insights into the model’s decision-making process and highlights the integration of information from multiple layers for more robust segmentation results.

**FIGURE 9:**
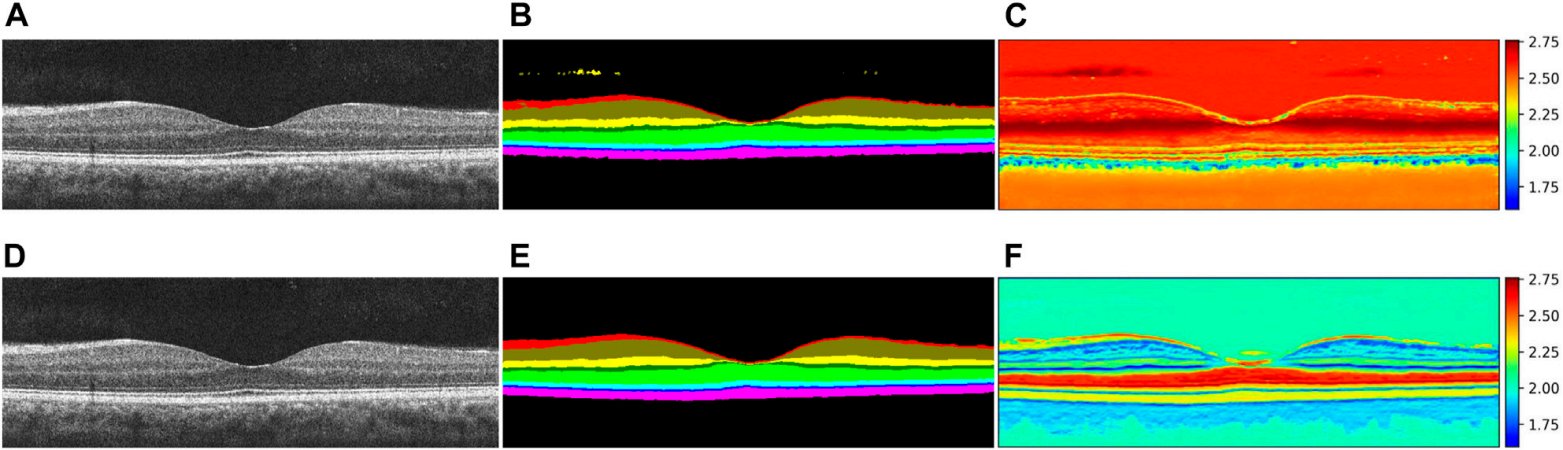
Uncertainty visualization of segmentation probabilities. **(A)** and **(D)** are the same retina layer graph, **(B)** and **(E)** are the segmentation result graphs for Attention U-Net and our method respectively, and **(C)** and **(F)** are two corresponding uncertainty probability graphs, respectively.

In our segmentation task, each pixel is associated with multiple category output probabilities. We use the entropy measure (Kapur and Kesavan, 1992) to quantify the uncertainty of the model’s output. The entropy is defined as follows:

$$H\left(X\right)=-\underset{i}{\sum }p\left(x_{i}\right)\log\left(p\left(x_{i}\right)\right),$$ (9)

where *p* (*x*_*i*_) denotes the output probability of category *x*_*i*_. Specifically, when a retinal image is processed by a segmentation model, each pixel is associated with *m* values (*m* represents the number of retinal layers segmented), and each value represents the output probability of each category. We translate these values so that the minimum value is greater than 0 and the sum of these *m* values is 1. The transformed values are *p* (*x*_*i*_), where $i\in \left(0,m\right]$ . The higher the probability of certain categories is, the lower the entropy is. The more uniform the probability of the categories is, the higher the entropy is. In Figure 9F, we observe that the entropy values of the GCL layer to the OPL layer, the OS layer, and the background region are relatively lower, indicating lower uncertainty of the model in these regions. However, in the ONL layer and macular region, there is a significant increasing trend of uncertainty. This trend may be attributed to the wider interval of pixel value distribution in this region, leading to a more uniform probability distribution output by the model, as depicted in Figure 9D. Interestingly, although the uncertainty is higher in the ONL layer and macular region, as shown in Figure 9E, the segmentation results in this region do not exhibit significant errors. This finding suggests that the occurrence of segmentation errors increases when the uncertainty reaches a certain level, beyond which the model struggles to provide accurate predictions. The analysis of uncertainty provides insights into the reliability and confidence of the model’s predictions. By examining the entropy values, we can identify regions with higher uncertainty, which may require further investigation or additional expert input to ensure reliable segmentation results.

### 5.4 Ablation studies

We perform ablation experiments on the NR206 dataset with the main objectives to verify 1) the impact of various feature backbones on the segmentation performance and 2) the role of the DE-CBAM module on the whole framework.**Impacts of feature backbones.** We compare ConvNeXt with Vgg16 (Simonyan and Zisserman, 2014), ResNet (He et al., 2016), Xception (Chollet, 2017), ViT (Dosovitskiy et al., 2020), and other mainstream backbones in the framework for DeepLabV3+ without the ASPP module. Table 4 shows that the ConvNeXt enables us to achieve a higher segmentation quality and outperforms the second-best place, ResNet152, by 0.7% on mIoU.**Impacts of DE-CBAM.** The experimental results are shown in Table 5. By using the DE-CBAM module, we can improve the performance over the CBAM module; specifically, we achieve a +3.5% Dice score on average. We observed improved performance of DE-CBAM by increasing the depth of the network. As described in Section 3.4, we do not change the internal structure of the CBAM and insert two consecutive depth-wise separable convolutions at its output, which improves the network’s utilization of the feature map.

**TABLE 4:** mIoU (%) of the segmentation results on the NR206 dataset by different mainstream backbones in the framework for DeepLabV3+ without the ASPP module. The best performance is in bold.

Vgg16	ResNet101	ResNet152	ViT16	ViT32	Xception	ConvNeXt-B
81.1	81.8	84.0	68.9	71.1	82.4	**84.7**

**TABLE 5:** Ablation experiment of the DE-CBAM in our framework performed by comparing the Dice score (%) on the NR206 dataset. The settings are marked with an asterisk (★), and the best performance on the two scales and four scales is in bold.

CBAM	DE-CBAM	NFL	GCL	INL	OPL	ONL	ELM + IS	OS	RPE	Average
**★**		86.2	91.8	85.8	74.4	89.5	91.8	86.9	95.3	88.0
	**★**	**89.4**	**96.0**	**90.4**	**82.4**	**95.4**	**92.7**	**87.8**	**96.1**	**91.5**

## 6 Conclusion

We presented a novel multi-scale end-to-end network for improved retinal layer segmentation in normal healthy human eyes. Our network incorporates a state-of-the-art attention module to efficiently utilize feature information and utilizes a ConvNeXt-based backbone for accurate segmentation. In addition, we provide a semantic segmentation dataset comprising 206 retinal layer OCT images of healthy human eyes, with each image annotated into nine classes. The dataset has an average percentage of annotated pixels in the retinal layer of 14.82%, excluding the background. This dataset is readily usable without requiring any pre-processing steps. We validate our approach on the NR206 dataset and a glaucoma dataset, demonstrating superior segmentation performance compared to other state-of-the-art methods. Our approach achieves an average Dice score of 91.3% and mIoU of 84.4% on the NR206 dataset and similar excellent performance on the glaucoma dataset. Furthermore, we evaluate our method on a DME dataset to demonstrate its generalization capability, achieving the best performance with a smaller number of parameters. In future research, we suggest exploring the application of retinal layer segmentation in the early detection of ophthalmic diseases. This field holds significant potential for improving disease diagnosis and treatment monitoring in ophthalmology.

## Data Availability

The raw data supporting the conclusion of this article will be made available by the authors, without undue reservation.

## References

[B1] Bhartiya S. Gadia R. Sethi H. S. Panda A. (2010). Clinical evaluation of optic nerve head in glaucoma. J. Curr. Glaucoma Pract. 4, 115–132. 10.5005/jp-journals-10008-1080

[B2] Chan A. Duker J. S. Ko T. H. Fujimoto J. G. Schuman J. S. (2006). Normal macular thickness measurements in healthy eyes using stratus optical coherence tomography. Archives Ophthalmol. 124, 193–198. 10.1001/archopht.124.2.193 PMC1941772 16476888

[B3] Chen C. Zhou K. Zha M. Qu X. Guo X. Chen H. (2021). An effective deep neural network for lung lesions segmentation from Covid-19 ct images. IEEE Trans. Industrial Inf. 17, 6528–6538. 10.1109/tii.2021.3059023 PMC8545014 37981911

[B4] Chen L.-C. Zhu Y. Papandreou G. Schroff F. Adam H. (2018). “Encoder-decoder with atrous separable convolution for semantic image segmentation,” in Proceedings of the European conference on computer vision, Germany, 06 October 2018 (ECCV), 801–818.

[B5] Chinn S. Swanson E. Fujimoto J. (1997). Optical coherence tomography using a frequency-tunable optical source. Opt. Lett. 22, 340–342. 10.1364/ol.22.000340 18183195

[B6] Chiu S. J. Allingham M. J. Mettu P. S. Cousins S. W. Izatt J. A. Farsiu S. (2015). Kernel regression based segmentation of optical coherence tomography images with diabetic macular edema. Biomed. Opt. express 6, 1172–1194. 10.1364/boe.6.001172 25909003 PMC4399658

[B7] Chollet F. (2017). “Xception: Deep learning with depthwise separable convolutions,” in Proceedings of the IEEE conference on computer vision and pattern recognition, USA, 17-19 June 1997 (IEEE), 1251–1258.

[B8] Chrástek R. Wolf M. Donath K. Niemann H. Paulus D. Hothorn T. (2005). Automated segmentation of the optic nerve head for diagnosis of glaucoma. Med. image Anal. 9, 297–314. 10.1016/j.media.2004.12.004 15950894

[B9] Dosovitskiy A. Beyer L. Kolesnikov A. Weissenborn D. Zhai X. Unterthiner T. (2020). An image is worth 16x16 words: Transformers for image recognition at scale. arXiv preprint arXiv:2010.11929

[B10] Dufour P. A. Ceklic L. Abdillahi H. Schroder S. De Dzanet S. Wolf-Schnurrbusch U. (2012). Graph-based multi-surface segmentation of oct data using trained hard and soft constraints. IEEE Trans. Med. imaging 32, 531–543. 10.1109/tmi.2012.2225152 23086520

[B11] Fercher A. F. Hitzenberger C. K. Kamp G. El-Zaiat S. Y. (1995). Measurement of intraocular distances by backscattering spectral interferometry. Opt. Commun. 117, 43–48. 10.1016/0030-4018(95)00119-s

[B12] Fernández D. C. Salinas H. M. Puliafito C. A. (2005). Automated detection of retinal layer structures on optical coherence tomography images. Opt. express 13, 10200–10216. 10.1364/opex.13.010200 19503235

[B13] Gholami P. Roy P. Parthasarathy M. K. Lakshminarayanan V. (2020). Octid: Optical coherence tomography image database. Comput. Electr. Eng. 81, 106532. 10.1016/j.compeleceng.2019.106532

[B14] Hatamizadeh A. Tang Y. Nath V. Yang D. Myronenko A. Landman B. (2022). Unetr: Transformers for 3d medical image segmentation. Proc. IEEE/CVF Winter Conf. Appl. Comput. Vis., 574, 584. 10.1109/WACV51458.2022.00181

[B15] He K. Zhang X. Ren S. Sun J. (2016). “Deep residual learning for image recognition,” in Proceedings of the IEEE conference on computer vision and pattern recognition, USA, 17-19 June 1997 (IEEE), 770–778.

[B16] He Y. Carass A. Solomon S. D. Saidha S. Calabresi P. A. Prince J. L. (2019). Retinal layer parcellation of optical coherence tomography images: Data resource for multiple sclerosis and healthy controls. Data brief 22, 601–604. 10.1016/j.dib.2018.12.073 30671506 PMC6327073

[B17] Huang D. Swanson E. A. Lin C. P. Schuman J. S. Stinson W. G. Chang W. (1991). Optical coherence tomography. Science 254, 1178–1181. 10.1126/science.1957169 1957169 PMC4638169

[B18] Kapur J. N. Kesavan H. K. (1992). Entropy optimization principles and their applications. Germany: Springer.

[B19] Karri S. Chakraborthi D. Chatterjee J. (2016). Learning layer-specific edges for segmenting retinal layers with large deformations. Biomed. Opt. express 7, 2888–2901. 10.1364/boe.7.002888 27446714 PMC4948638

[B20] Lam F. Longnecker M. (1983). A modified wilcoxon rank sum test for paired data. Biometrika 70, 510–513. 10.1093/biomet/70.2.510

[B21] Le N. Q. K. Huynh T.-T. (2019). Identifying snares by incorporating deep learning architecture and amino acid embedding representation. Front. Physiology 10, 1501. 10.3389/fphys.2019.01501 PMC6914855 31920706

[B22] Le N. Q. K. (2021). Potential of deep representative learning features to interpret the sequence information in proteomics. Proteomics 22, e2100232. 10.1002/pmic.202100232 34730875

[B23] Li J. Jin P. Zhu J. Zou H. Xu X. Tang M. (2021). Multi-scale gcn-assisted two-stage network for joint segmentation of retinal layers and discs in peripapillary oct images. Biomed. Opt. Express 12, 2204–2220. 10.1364/boe.417212 33996224 PMC8086482

[B24] Li Q. Li S. He Z. Guan H. Chen R. Xu Y. (2020). Deepretina: Layer segmentation of retina in oct images using deep learning. Transl. Vis. Sci. Technol. 9, 61. 10.1167/tvst.9.2.61 33329940 PMC7726589

[B25] Liu Z. Lin Y. Cao Y. Hu H. Wei Y. Zhang Z. (2021). “Swin transformer: Hierarchical vision transformer using shifted windows,” in Proceedings of the IEEE/CVF International Conference on Computer Vision, USA, 11-17 Oct. 2021 (IEEE), 10012–10022.

[B26] Liu Z. Mao H. Wu C.-Y. Feichtenhofer C. Darrell T. Xie S. (2022). “A convnet for the 2020s,” in Proceedings of the IEEE/CVF Conference on Computer Vision and Pattern Recognition, USA, 19-20 June 2022 (IEEE), 11976–11986.

[B27] London A. Benhar I. Schwartz M. (2013). The retina as a window to the brain-from eye research to cns disorders. Nat. Rev. Neurol. 9, 44–53. 10.1038/nrneurol.2012.227 23165340

[B28] Long J. Shelhamer E. Darrell T. (2015). Fully convolutional networks for semantic segmentation. IEEE Trans. Pattern Analysis Mach. Intell. 39, 640–651. 10.1109/tpami.2016.2572683 27244717

[B29] Min S. Lee B. Yoon S. (2017). Deep learning in bioinformatics. Briefings Bioinforma. 18, 851–869. 10.1093/bib/bbw068 27473064

[B30] Oktay O. Schlemper J. Folgoc L. L. Lee M. Heinrich M. Misawa K. (2018). Attention u-net: Learning where to look for the pancreas. arXiv preprint arXiv:1804.03999

[B31] Pavkov M. E. Harding J. L. Chou C.-F. Saaddine J. B. (2019). Prevalence of diabetic retinopathy and associated mortality among diabetic adults with and without chronic kidney disease. Am. J. Ophthalmol. 198, 200–208. 10.1016/j.ajo.2018.10.019 30691612

[B32] Ronneberger O. Fischer P. Brox T. (2015). “U-net: Convolutional networks for biomedical image segmentation,” in International Conference on Medical image computing and computer-assisted intervention, germany, 18-22 September (Springer), 234–241.

[B33] Roy A. G. Conjeti S. Karri S. P. K. Sheet D. Katouzian A. Wachinger C. (2017). Relaynet: Retinal layer and fluid segmentation of macular optical coherence tomography using fully convolutional networks. Biomed. Opt. express 8, 3627–3642. 10.1364/boe.8.003627 28856040 PMC5560830

[B34] Simonyan K. Zisserman A. (2014). Very deep convolutional networks for large-scale image recognition. *arXiv preprint arXiv:1409.1556*

[B35] Srinivasan P. P. Kim L. A. Mettu P. S. Cousins S. W. Comer G. M. Izatt J. A. (2014). Fully automated detection of diabetic macular edema and dry age-related macular degeneration from optical coherence tomography images. Biomed. Opt. express 5, 3568–3577. 10.1364/boe.5.003568 25360373 PMC4206325

[B36] Stitt A. W. Curtis T. M. Chen M. Medina R. J. McKay G. J. Jenkins A. (2016). The progress in understanding and treatment of diabetic retinopathy. Prog. Retin. eye Res. 51, 156–186. 10.1016/j.preteyeres.2015.08.001 26297071

[B37] Tian J. Varga B. Tatrai E. Fanni P. Somfai G. M. Smiddy W. E. (2016). Performance evaluation of automated segmentation software on optical coherence tomography volume data. J. Biophot. 9, 478–489. 10.1002/jbio.201500239 PMC5025289 27159849

[B38] Ueda E. Hirabayashi N. Ohara T. Hata J. Honda T. Fujiwara K. (2022). Association of inner retinal thickness with prevalent dementia and brain atrophy in a general older population: The hisayama study. Ophthalmol. Sci. 2, 100157. 10.1016/j.xops.2022.100157 36249677 PMC9559916

[B39] Vinogradova K. Dibrov A. Myers G. (2020). Towards interpretable semantic segmentation via gradient-weighted class activation mapping (student abstract). Proc. AAAI Conf. Artif. Intell. 34, 13943–13944. 10.1609/aaai.v34i10.7244

[B40] Wang Y. Yang T. Huang W. (2020). “Limited-angle computed tomography reconstruction using combined fdk-based neural network and u-net,” in 2020 42nd Annual International Conference of the IEEE Engineering in Medicine & Biology Society (EMBC), USA, 20-24 July 2020 (IEEE), 1572. 10.1109/EMBC44109.2020.9176040 33018293

[B41] Woo S. Park J. Lee J.-Y. Kweon I. S. (2018). “Cbam: Convolutional block attention module,” in Proceedings of the European conference on computer vision (ECCV), New York, October 23-27 (IEEE), 3.

[B42] Yu C. Wang J. Peng C. Gao C. Yu G. Sang N. (2018). “Bisenet: Bilateral segmentation network for real-time semantic segmentation,” in Proceedings of the European conference on computer vision, Germany, 23-27 October (ECCV), 325–341.

[B43] Zhang Y. Li M. Yuan S. Liu Q. Chen Q. (2021). Robust region encoding and layer attribute protection for the segmentation of retina with multifarious abnormalities. Med. Phys. 48, 7773–7789. 10.1002/mp.15315 34716932

